# Increase in Comforting Behavior (Allogrooming) During Social Interaction in Male Mice Deficient for the *Slp* Gene of Complement Component C4

**DOI:** 10.3390/brainsci16010081

**Published:** 2026-01-07

**Authors:** Yasuhiko Yamamoto, Anpei Zhang, Anna A. Shabalova, Ai Harashima, Kyota Fujita, Teruko Yuhi, Yu Oshima, Pinyue Fu, Sei-ichi Munesue, Kana Minami, Kazuhiro Higashida, Hirokazu Kumazaki, Chiharu Tsuji, Haruhiro Higashida

**Affiliations:** 1 Department of Biochemistry and Molecular Vascular Biology, Graduate School of Medical Sciences, Kanazawa University, Kanazawa 920-8640, Japan; yasuyama@med.kanazawa-u.ac.jp (Y.Y.); cantabile.6102@gmail.com (Y.O.);; 2 Department of Basic Research on Social Recognition and Memory, Research Center for Child Mental Development, Kanazawa University, Kanazawa 920-8640, Japan

**Keywords:** prosocial behavior, comforting behavior, complement component C4, C4a, oxytocin release, hypothalamus, mouse

## Abstract

**Background**: Oxytocin (OT) is a nonapeptide hormone produced in the hypothalamus, released into the brain and peripheral circulation, and plays a key role in social behavior. Recent studies indicate that complement component C4a is an OT-binding protein, which modulates plasma OT concentrations in mice. However, the role of C4a is unclear as to whether it contributes to consolation behavior. **Methods**: Social behavior, especially allogrooming, which is a form of empathy that depends on detecting the emotional states of others, was measured in wild-type or *C4a/Slp* knockout (*Slp*^−/−^) male mice. **Results**: Observer mice of both genotypes exhibited comforting (allogrooming) behavior toward a cage-mate demonstrator during reunion after brief isolation of the demonstrator mice. When demonstrator mice experienced body restraint stress during isolation, the allogrooming behavior was significantly increased in both genotypes, with a markedly greater increase in *Slp*^−/−^ than in *Slp*^+/+^ mice. Allogrooming behavior in observer *Slp*^−/−^ mice was significantly suppressed by an OT receptor antagonist. Furthermore, immunohistochemical analysis revealed that activation was significantly elevated in OT-positive hypothalamic neurons in observer *Slp*^−/−^ mice that interacted with stressed demonstrator mice. OT release from the isolated hypothalamus, stimulated via CD38 and TRPM2 channel activation, was greater in *Slp*^−/−^ mice than in *Slp*^+/+^ mice. **Conclusions**: Our results highlight that the data are consistent with a potential role for C4a in modulating neural circuits, possibly via its peripheral action on OT bioavailability. Direct evidence for C4a’s action within the brain remains a hypothesis for future investigation, for example, via site-specific manipulations.

## 1. Background

Oxytocin (OT) and OT receptors play crucial roles in regulating social behavior [1,2,3,4,5,6,7,8,9,10,11,12,13,14,15,16,17]. Mice with deletion of OT or OT receptor genes exhibit impaired social behaviors [3,4,18,19,20,21,22]. Moreover, deletion of Cluster of Differentiation 38 (CD38) in mice results in social behavior deficits in models of autism spectrum disorder, because CD38 is essential for OT-induced OT release in hypothalamic oxytocinergic neurons [7,12,23,24,25,26,27]. The NAD metabolite cyclic ADP-ribose, generated by CD38’s ADP-ribosyl cyclase activity, facilitates OT release in the brain and elevates brain OT concentrations [27]. Recently, the receptor for advanced glycation end-products (RAGE) was identified as an OT-binding partner in the blood. In neurovascular endothelial cells, RAGE mediates OT transport across the blood–brain barrier (BBB), thereby increasing central OT concentrations. Furthermore, social behavior is impaired in RAGE knockout mice, demonstrating that RAGE delivers OT to the brain, where it subsequently modulates social behavior [28].

Recently, a novel OT-binding protein in human serum was identified by capturing potential OT-binding proteins using a specially designed click chemistry probe [28]. Proteomic analysis revealed that this candidate protein was complement component C4a. C4a is a proteolytic fragment cleaved from C4, typically generated upon activation of the immune complement system [29,30]. Notably, both high and low levels of *C4a* copy number in neurons or astrocytes are associated with schizophrenia [31,32] and Alzheimer’s disease [33], respectively, and C4 deficiency, and consequently C4a deficiency, is strongly linked to autoimmune diseases [30]. The high-affinity binding between C4a and OT indicates a specific interaction of C4a with OT in human blood [28]. Therefore, C4a appears to play a critical role in regulating plasma concentrations of free OT equilibrated with the C4a-bound form of OT [28], potentially altering OT transport to the brain from the circulation via endothelial RAGE [29,34]. An increase in plasma OT concentration following intraperitoneal OT administration was observed in *C4a* knockout mice, in which the sex-limited protein (*Slp*) gene, the mouse equivalent of the human *C4* gene [35,36], was deleted in males (*Slp*^−/−^) [28]; consequently, *Slp*^−/−^ mice may have enhanced brain OT supply from the circulation [34].

In studies of social behavior, monogamous mice have been shown to perform comforting behaviors toward demonstrator mice experiencing emotional stress induced by external stressors, such as body restraint [37,38,39]. One form of this consolation behavior is termed allogrooming [40]. Allogrooming behavior is blocked by the administration of an OT receptor antagonist [41], suggesting that co-housed cage mates exhibit consolation-related behaviors in an OT signal-dependent manner [41,42]. Nevertheless, the precise roles of the OT system and the underlying molecular mechanisms in the hypothalamus governing allogrooming toward distressed conspecifics remain unclear.

In the present study, we aimed to examine whether *Slp*^−/−^ male mice of the ICR genetic background, which exhibited increased social contact in a previous study [28], can recognize the emotional state of their cage mates and display comforting behavior, particularly whether knockout mice show enhanced allogrooming toward distressed conspecifics, although the ICR mouse is not a monogamous strain [7,27]. Furthermore, we sought to investigate the involvement of hypothalamic oxytocinergic neurons during allogrooming by assessing c-Fos protein expression, a marker of neuronal activation, and OT release via the CD38 and TRPM2 channels, components of the OT-induced OT release system [43].

## 2. Methods

### 2.1. Animals

Eight-week-old ICR male and female mice were obtained from Japan SLC Inc. (Hamamatsu, Japan) through a local distributor (Sankyo Laboratory Service Corp., Toyama, Japan) and served as controls for the *C4a/Slp* knockout (*Slp*^−/−^) mice. *C4a* knockout mice were generated using the CRISPR/Cas9 system, as previously described [28]. Briefly, the mouse homolog of the human *C4B(H)* gene, the *Slp* gene [44], was disrupted, resulting in *Slp*^−/−^ mice carrying the expected knockout allele as confirmed by Southern blot analysis [28]. Plasma C4a concentrations in *Slp*^−/−^ mice were approximately half of those in wild-type mice, reflecting the presence of an additional mouse *C4A* gene. *Slp*^−/−^ mice were maintained through the crossbreeding of homozygous mutant animals. Because the expression of the *Slp* gene is regulated by the androgen-responsive element in males, behavioral analyses were conducted exclusively in male mice in the present study.

Four mice were housed in one cage during weaning in same-sex groups that were kept in the animal center under standard conditions (24 °C; 12/12-h light/dark cycle with lights on at 8:45 a.m.) with food and water ad libitum. Afterward, two males of the same litter were housed together as cage mates. The wild-type and knockout offspring were kept with their biological mothers

All animal experiments were approved by the Committee on Animal Experimentation of Kanazawa University and conducted in accordance with the Fundamental Guidelines for Proper Conduct of Animal Experimentation and Related Activities in Academic Research Institutions, under the jurisdiction of the Ministry of Education, Culture, Sports, Science and Technology of Japan.

### 2.2. Open Field Test

**Figure 1:**
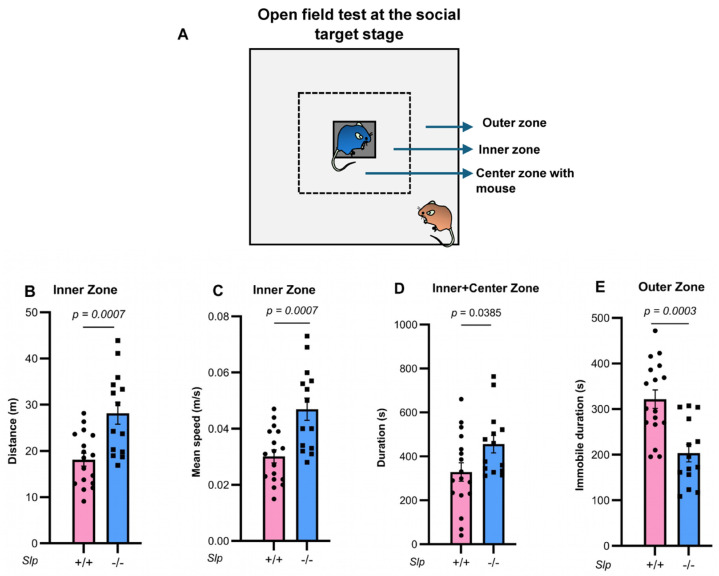
Open field tests at the social target stage. The dotted square, not present in the actual test field, represents the inner zone. (**A**). Two unfamiliar, experimentally naïve, age-matched, and weight-matched *Slp*^+/+^ or *Slp*^−/−^ male mice were placed in a meshed cage at the center zone or the outer zone of the open field. Distance traveled (**B**) and mean speed (**C**) in the inner zone of mice between the same genotypes. (**D**). Time spent in direct interaction with the target mouse in the meshed cage at the center (grey). (**E**). Immobilized time in the outer zone. *p* values calculated using the Student’s *t*-test. A dot represents an experimental result in one mouse.

**Figure 2:**
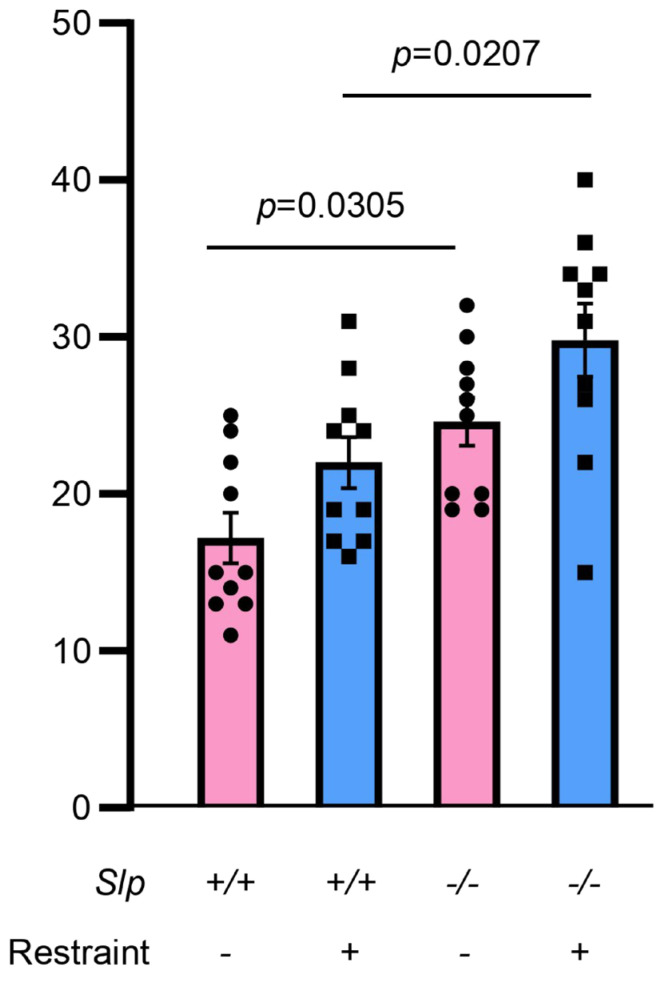
Frequency of social sniffing during direct interaction with the target mouse in the meshed cage at the center. An observer mouse of *Slp*^+/+^ or *Slp*^−/−^ mice interacted with a demonstrator mouse in the open field test. Demonstrators received no or body restraint before the behavior test. A dot represents an experimental result in one mouse.

ICR wild-type (*Slp*^+/+^) and *Slp*^−/−^ male mice were assessed for locomotor activity and anxiety-related behaviors using the open field test, as previously described [45]. A square open field apparatus (600 × 600 × 200 mm) was used, with the floor covered by polypropylene sheets. An inner arena (300 × 300 mm) was demarcated within the field. A small, meshed cage containing a male mouse served as a social target and was positioned in the central arena (Figure 1A). Overall activity within the open field was monitored for 10 min (Figure 1 and Figure 2) using a digital video system and ANY-maze software (Version 2010) (Stoelting Co., Wood Dale, IL, USA). Time spent and distance traveled in the outer, inner, and inner plus central arenas were quantified automatically. After each trial, the apparatus was cleaned sequentially with a damp towel, 1% sodium hypochlorite, and 70% ethanol to remove olfactory cues and prevent confounding social behavior responses [45].

### 2.3. Allogrooming Test

**Figure 3:**
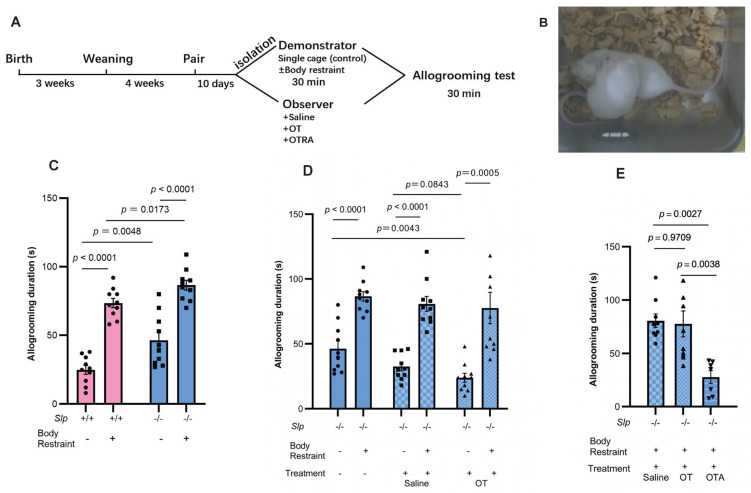
Allogrooming behavior tests. (**A**) Experimental schedule showing co-housing, isolation with or without body restraint, and allogrooming tests for 30 min during reunion. (**B**) Representative allogrooming behavior. An observer mouse holds and licks the back of a demonstrator mouse. (**C**) Allogrooming duration of the observer *Slp*^+/+^ and *Slp*^−/−^ mice during 30 min reunion with *Slp*^+/+^ and *Slp*^−/−^ demonstrator mice after isolation with (+) or without (−) body restraint stress. Effect of intraperitoneal OT (30 ng/mouse, (**D**)) or atosiban (20 μg/mouse; (**E**)) on allogrooming duration in observer *Slp*^+/+^ and *Slp*^−/−^ mice. OT, atosiban (OTA), or saline were administered to observer mice 20 min before behavior tests. A dot represents an experimental result in one mouse.

**Figure 4:**
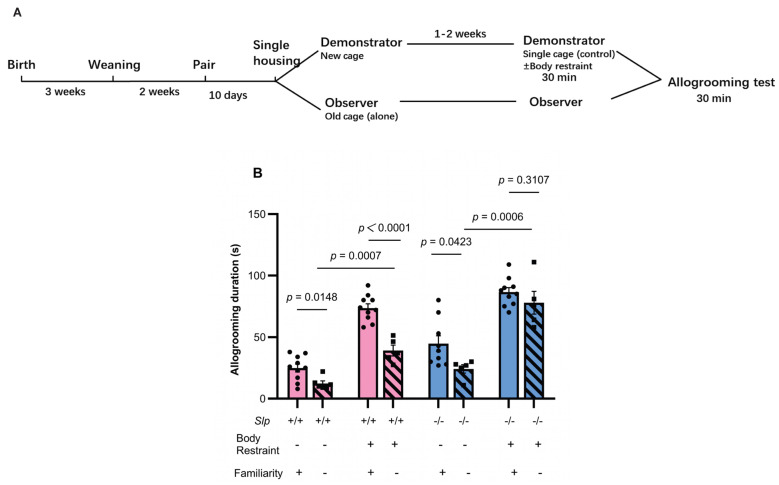
Effects of single housing on allogrooming in previously co-housed pairs. (**A**) Experimental schedule of cage mate mice; the design is essentially identical to Figure 3A, except for the single housing period. (**B**) Effects of familiarity on allogrooming behavior of the observer *Slp*^+/+^ and *Slp*^−/−^ mice during 30 min upon reunion after 30-min isolation with (+) or without (−) body restraint stress in demonstrator mice. Allogrooming duration was compared between previously cohoused (familiarity, +) pairs and single-housed pairs after prior cohousing (familiarity, −). A dot represents an experimental result in one mouse.

The allogrooming test for *Slp*^+/+^ and *Slp*^−/−^ male mice was conducted as previously described [37,41]. Mouse litter mates remained with their mothers in nursing cages for 3 weeks postnatal. Following weaning, 4 mice were housed per cage for an additional 4 weeks. Two young adult males of similar body weights were randomly selected and either paired and co-housed for 10 days or individually housed for 10 days (Figure 3A and Figure 4A).

On the day of testing, paired male mice that had cohabited for the specified periods were randomly assigned as either an observer or a demonstrator because there was no or little difference in apparent shapes. One of them without any special selection was selected from the two as the demonstrators and subjected to either simple isolation in a novel cage (control) or body restraint, as shown in Figure 3 and Figure 4. In the control group, the demonstrator mouse was removed from the home cage and placed into a clean cage with wooden bedding for 30 min. Afterward, it was returned to reunite with its cage mate (observer). In the restraint stress group, the demonstrator was separated from its cage and restrained for 30 min in a 50-mL polystyrene tube with small perforations at the tail and mouth ends to permit ventilation. Throughout all experiments, demonstrator and observer mice were housed in non-transparent aluminum cages located at least 30–50 cm apart. Under these conditions, distressing events were unable to be observed. However, distressing signals from the stressed demonstrator might be transmitted via visual, olfactory and auditory channels during reunion, consistent with previous reports [41,46], although there was no direct evidence.

Following the 30-min restraint period, the demonstrator mouse was released from the tube and returned to the home cage for reunion. Then, the observer mouse observed the demonstrator for 30 min. The behavior was recorded using a digital video system (ANY-maze software). All mice used in these behavioral experiments were experimentally naïve, same-sex, age-matched, of the same genotype, and familiar with each other unless otherwise specified.

Typical allogrooming was defined as investigative behavior predominantly directed toward the dorsal flank, neck, and head of the conspecific, accompanied by rhythmic head movements, during which the demonstrator mouse showed head bobbing indicative of licking motions. Close investigation was defined as a demonstrator mouse orienting its snout toward another mouse and positioning itself within half a head length of the other mouse, consistent with previous descriptions [46], (Figure 3B; Supplementary Movies). Grooming directed toward the genitals, anogenital region, or tail, or occurring during mating bouts, was classified as genital/sexual grooming and excluded from analysis. Affiliative behaviors, including body-directed touches and licking of the demonstrator, were included. Aggressive allogrooming events were rare in *Slp*^−/−^ male mice and were not quantified. Behavioral scoring was performed by two independent researchers, one of whom was blind to the experimental conditions.

To assess the effects of OT or the OT receptor antagonist atosiban, observer mice received intraperitoneal injections of OT (30 ng/mouse) or atosiban (20 μg/mouse) 30 min or 20 min prior to the reunion, respectively [41].

### 2.4. Immunohistochemistry

**Figure 5:**
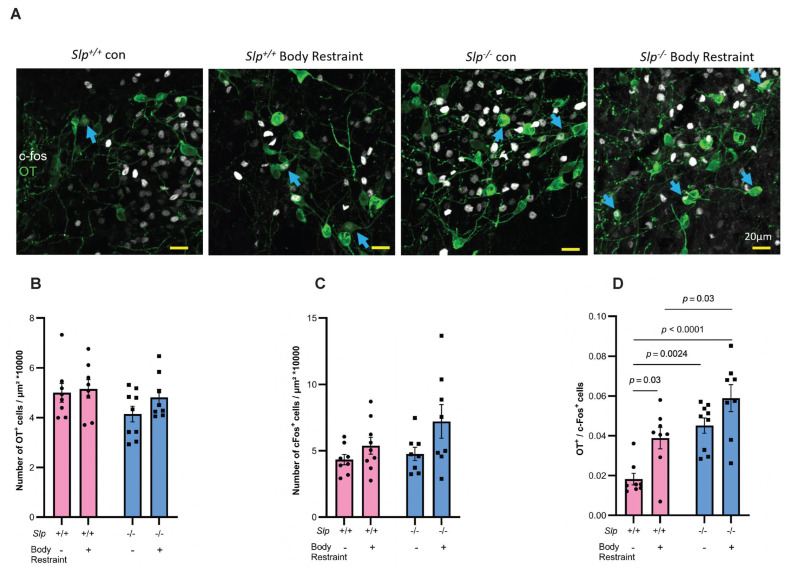
Immunostaining of the hypothalamic paraventricular nucleus of observer male mice. (**A**) Observer mice interacted with cage-mate demonstrators for 30 min and were sacrificed 45–50 min later. Images illustrate c-Fos (white) and OT (green) immunoreactivity. Blue arrows indicate c-Fos-positive nuclei within OT immunoreactive neurons. Number of OT-positive cells (**B**) or c-Fos-positive cells (**C**) in *Slp*^+/+^ and *Slp*^−/−^ mice with or without body restraint. (**D**). Number of co-staining cells. *p* values are described in the figure. A dot represents an experimental result in one mouse.

Following the 30-min allogrooming interaction, observer male mice were retained in their home cages for an additional 30 min to allow for c-Fos protein induction [47]. Observer mice were anesthetized with isoflurane, and individuals exhibiting near-average allogrooming frequency within each experimental group were selected for analysis. The mice were intracardially perfused with cold phosphate-buffered saline (PBS) followed by cold 4% paraformaldehyde (PFA) in PBS. The anesthesia and perfusion procedure required approximately 15–20 min to complete. Brains were removed and postfixed in 4% PFA overnight at 4 °C. Brain tissue was then divided into 2–4 larger blocks, which were sectioned into 40-μm-thick slices using a microtome. The sections were rinsed in PBS for 5 min, three times, then permeabilized in washing buffer (0.3% Triton X-100 in PBS) (Nacalai Tesque, Kyoto, Japan) for 20 min followed by preincubation in blocking solution containing 3% bovine serum albumin and 0.3% Triton X-100 in PBS for 1 h [47]. The slices were subsequently incubated with a mouse anti-OT monoclonal antibody (1:500; PS38, MABN844, Merck Millipore, Merck KGaA, Darmstadt, Germany) and/or a rabbit anti-c-Fos polyclonal antibody (1:100,00, 226008, Synaptic Systems, Göttingen, Germany) in blocking solution for 48 h at 4 °C. Following three washes with buffer, sections were incubated with a goat anti-rabbit IgG secondary antibody conjugated to Alexa Fluor 488 (1:200, A-11001, Thermofisher Scientific, Waltham, MA, USA) and goat anti-rabbit IgG antibody conjugated with Alexa Fluor 594 (1:200, A-11012, Thermofisher Scientific) in blocking solution for 1 h at room temperature. The sections were then rinsed with PBS for 5 min, three times, and mounted with PermaFluor Aqueous Mounting Medium (TA-030-FM, Thermo Scientific, Kalamazoo, MI, USA). Fluorescence images were acquired using a BZ-X810 all-in-one microscope (Keyence, Osaka, Japan), as shown in Figure 5.

The number of c-Fos immunoreactive nuclei in each brain section was quantified using ImageJ (NIH, Bethesda, MD, USA). Brain structures were anatomically identified according to the atlas of Franklin and Paxinos (1997) [48]. Sections used for quantification were selected from bregma levels between −0.82 mm and −0.94 mm along the anterior–posterior (AP) axis. Quantification of c-Fos- and/or OT–immunopositive cells was performed within the PVN, which served as the counting area. PVN boundaries were manually delineated based on the distribution of OT-positive signal, and c-Fos and/or OT counts were normalized to the corresponding PVN area. The counts were averaged from three brain sections to obtain values for each mouse.

### 2.5. OT Release from the Isolated Hypothalamus

**Figure 6:**
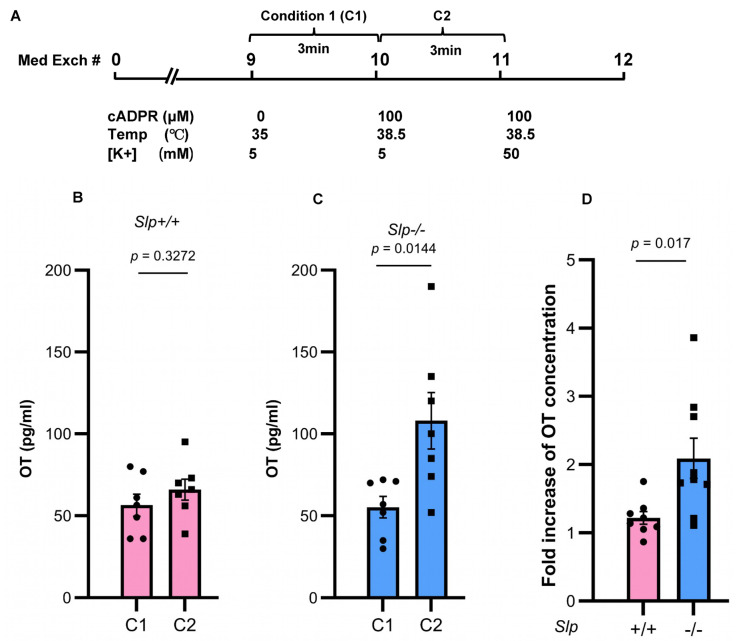
Concentrations of OT released from the whole hypothalamus isolated from *Slp*^+/+^ and *Slp*^−/−^ male mice in 0.4 mL incubation medium for 3 min. (**A**) Schedule of incubation. The isolated hypothalamus was washed by changing the incubation medium 9 times. (**B**) Concentrations of OT in dishes with control conditions (C1; 0 mM cADPR at 35 °C) or stimulated conditions (C2; 100 μM cADPR at 38.5 °C) released from the *Slp*^+/+^ hypothalamus. (**C**) Concentrations of OT in dishes with control conditions (C1; 0 mM cADPR at 35 °C) or stimulated conditions (C2; 100 μM cADPR at 38.5 °C) released from the *Slp*^−/−^ hypothalamus. Concentrations of OT in dishes with 100 mM cADPR incubated at 38.5 °C. (**D**) Fold increase of OT release (C2/C1) under combined cADPR and heat stimulation. *p* = 0.0170. Results obtained from wild-type (pink) and knockout (blue) mice. A dot represents an experimental result in one mouse.

Mice were anesthetized with isoflurane, and one whole hypothalamus was dissected and placed in a single well of a 24-well plate containing 0.4 mL of Locke’s solution (in mM: NaCl, 140; KCl, 5; MgCl_2_, 1.2; CaCl_2_, 2.2; glucose, 10; HEPES, 10; and 0.01% bovine serum albumin), adjusted to pH 7.25 with Tris-HCl and maintained in a water bath at 35 °C [43,47]. The incubation medium was refreshed 10 times at 3-min intervals to stabilize basal OT release. Following the 9th replacement, aliquots were collected after a 3-min incubation with or without 100 μM cyclic ADP-ribose (cADPR, Sigma, St. Loiuis, MO, USA), and the temperature increased from 35 to 38.5 °C using two separate incubators (Figure 6A).

### 2.6. Elevated Plus Maze Test

The elevated plus maze (EPM) test was conducted for 5 min under dim illumination (20 lx), as described previously [49]. The time spent and frequency of entries into each arm were automatically recorded and analyzed using the camera-assisted ANY-maze software. Time spent in the open arms was used as a measure of anxiety-related behavior.

### 2.7. Tail Suspension Test

The tail suspension test (TST) was performed as previously described [45]. Behavior was recorded for 6 min using video capture and analyzed with ANY-maze software. Total immobility time during the final 4 min of the 6-min session was used as an index of behavioral despair.

### 2.8. Experimental Design

During weaning, there were no apparent differences in mice, so we chose mice randomly without any special criteria. There was essentially no apparent difference in paired mice; the two mice in a paired cage were randomly selected as either a demonstrator or observer. Mice from groups to individual housing for 10 days before testing to lose familiarity from birth were also randomly selected. Every behavioral procedure was usually carried out between 1:00 and 6:00 p.m. All subjects were experimentally naïve. Mice were lightly anesthetized with isoflurane and euthanized by cervical dislocation following the completion of behavioral tests. Then, for the next experiment, groups of mice were prepared. Behavior was video-taped and scored by a trained blind rater (PY. F.) and non-blind rater (AP. Z.). When the scores of the two raters displayed great discrepancy, the video was re-inspected by both raters and discussed. Inter-rater reliability was >90%. In the present study, an a priori power analysis was not conducted. Instead, before designing the experiments, we reviewed previous reports employing similar experimental paradigms and confirmed that the planned sample size would be sufficient for the intended statistical analyses [24,28].

## 3. Statistics

Statistical analysis was performed using Prism software (GraphPad Prism 9.3.1; San Diego, CA, USA). For comparisons between two groups, a two-sided independent *t*-test was used. For comparisons of more than two groups, one-way analysis of variance (ANOVA) followed by Fisher’s least significant difference post hoc test was applied. Two-way ANOVA with Fisher’s least significant difference post hoc test was used for analyses involving two independent factors. Data are presented as the mean ± standard error of the mean unless otherwise indicated. Statistical significance was defined as *p* < 0.05. All individual data points represent biological replicates. Statistical results are summarized in Supplementary Table S1.

## 4. Results

### 4.1. Social Behavior in the Open Field Test

Behavior in the open field test was carefully reexamined in two genotypes of male mice (Figure 1). Upon exposure to a novel environment in the open field, the time spent in the inner zone was significantly increased in *Slp*^−/−^ mice, consistent with a previous report [28]. Furthermore, both the distance traveled (Figure 1B) and mean speed (Figure 1C) of *Slp*^−/−^ mice in the inner zone were higher than those of *Slp*^+/+^ mice (and *p* = 0.0007, for both). Time in the central zone with increased licking frequency when a male mouse was placed in a meshed small cage was significantly greater in *Slp*^−/−^ mice than in *Slp*^+/+^ mice (*p* = 0.0385; Figure 1D). In the outer zone, *Slp*^−/−^ mice exhibited reduced exploratory activity [28] and decreased immobility (Figure 1E, *p* = 0.0003). In addition, the frequency of social interaction or sniffing to demonstrator mice with or without body restraint was higher in *Slp*^−/−^ mice than *Slp*^+/+^ in the center zone (Figure 2; *p* = 0.0305, *p* = 0.0207, respectively). These results suggest that *Slp*^−/−^ male mice display higher levels of social interactive behavior toward social target mice.

### 4.2. C4a Knockout Mice Display Increased Comforting Behavior (Allogrooming)

To assess prosocial behavior, we further examined whether *Slp*^−/−^ male mice exhibited increased empathy toward their cage-mate mice using same-sex littermates. A cage mate (observer) mouse that had cohabited with another (demonstrator) mouse (shown in Figure 3A) displayed comforting behavior, known as allogrooming, toward the demonstrator [37,40,41]. Typical animal postures of allogrooming during 30 min upon reunion after isolation in new cages for 30 min (control condition) are shown in Figure 3B. *Slp*^−/−^ observer mice displayed a markedly longer allogrooming duration of 46.2 ± 2.8 s; whereas *Slp*^+/+^ observer male mice showed 25.1 ± 1.3 s (*n* = 10 each, *p* = 0.0048; Figure 3C no body restraint).

Allogrooming behavior in observer mice is known to persist longer toward distressed demonstrators [37,40,41]. Therefore, the demonstrator mice were isolated but confined in a small tube (body restraint stress), which was placed in a new, non-transparent cage. The allogrooming behavior of observer mice in both genotypes toward the cage mates (demonstrators) that underwent 30 min of body restraint stress during isolation was significantly increased during reunion in the old cage. This pronounced increase in allogrooming duration toward distressed demonstrators was observed in *Slp*^+/+^ mice (72.5 ± 2.5 s) and *Slp*^−/−^ mice (90.0 ± 3.7 s) (Figure 3C). This allogrooming duration of observers in both genotypes was significantly longer than that toward non-distressed (only isolated) cage mates (*p* < 0.0001). Furthermore, in tests of allogrooming toward distressed cage mates, *Slp*^−/−^ observer mice exhibited a longer allogrooming duration than the *Slp*^+/+^ observer mice (*p* = 0.0173).

To elucidate the role of OT in the observed enhancement of behavioral responses in *Slp*^−/−^ mice, intraperitoneal OT (30 ng/mouse) was administered to observer *Slp*^−/−^ mice 20 min prior to the behavioral testing. OT treatment resulted in a shorter allogrooming duration of observers to control demonstrators (only isolation). However, the allogrooming behavior toward distressed demonstrator mice with or without OT administration remained comparably and significantly prolonged (*p* = 0.0005; *p* = 0.0001, respectively; Figure 3D).

In contrast, the effects of an OT receptor antagonist (100 μM atosiban) on allogrooming were evaluated. The antagonist was administered to *Slp*^−/−^ observer mice 20 min before reunion with the demonstrators. OT receptor blockade markedly reduced the allogrooming duration (24 s, approximately one third of the control value; *p* = 0.0038; Figure 3E).

As control experiments, allogrooming behavior was examined between non-familiar cage mates (littermates housed separately) under nearly identical conditions, except for separate housing for 10 days before the encounter tests (Figure 4A). First, a similar tendency in allogrooming duration, as shown in Figure 3, was confirmed between familiar cage mates of *Slp*^+/+^ and *Slp*^−/−^ observer male mice, regardless of demonstrator restraint. Decreases in allogrooming duration arising from single housing were significant in *Slp*^+/+^ observer mice: allogrooming duration in non-familiar pairs of *Slp*^+/+^ mice was significantly reduced compared with familiar pairs (*p* = 0.0148 and *p* < 0.0001, respectively) (Figure 4B). On the other hand, in *Slp*^−/−^ mice, the reduction in allogrooming in non-familiar pairs was less pronounced (*p* = 0.0423 for no restraint; *p* = 0.3107 for restraint; Figure 4B). These results indicate that cohabitation (familiarity) is a critical factor in prosocial allogrooming in *Slp*^+/+^ mice to a greater extent than in *Slp*^+/+^ mice and suggest the specificity of impairment or hypersensitivity to the emotional clues displayed by the *Slp*^−/−^ demonstrator mice.

### 4.3. Activation of Oxytocin-Positive Hypothalamic Neurons During Allogrooming

Based on the above-mentioned findings, we further investigated hypothalamic neuron activation during allogrooming using immunohistochemical methods. Histochemical analysis was performed 45–50 min after the allogrooming experiments to allow for c-Fos protein induction (Figure 5A–D). The number of c-Fos positive cells in the hypothalamus of observer mice of both *Slp*^−/−^ and *Slp*^−/−^ genotypes after interaction with demonstrator mice, with or without body restraint, did not show significant differences (Figure 5C). Similarly, there were minimal differences in the number of OT-positive cells in the hypothalamus among the four groups of mice (Figure 5B). However, a significant difference was observed in the number of c-Fos-positive cells among the OT-positive cells (Figure 5A,D). Moreover, co-staining of c-Fos and OT was significantly higher in *Slp*^+/+^ mice interacting with distressed demonstrators than in those interacting with control demonstrators (*p* < 0.0300, F _(1, 29)_ = 23.18). *Slp*^−/−^ mice that interacted with non-restrained demonstrators exhibited significantly higher OT^+^ and c-Fos^+^ cell number (*p* = 0.0024). The number of such cells was the highest in the *Slp*^−/−^ mice that interacted with restraint demonstrators (*p* = 0.0024 or 0.0001, F _(1, 29)_ = 12.40). These findings suggest that OT neurons were more strongly activated during allogrooming behavior in *Slp*^−/−^ mice, indicating that OT signaling in the hypothalamus of *Slp*^−/−^ mice is more readily activated in response to social stimuli.

### 4.4. Increased Release of OT from the Isolated Hypothalamus

We investigated the molecular mechanisms within the hypothalamus of male observer mice that underlie the enhanced allogrooming behavior toward distressed demonstrator mice. To explore this, we measured stimulation-induced OT release from an isolated hypothalamus incubated in 0.4 mL of culture medium [43]. OT is known to be released through the activation of CD38, NAD-metabolizing enzymes, and the temperature-sensitive TRPM2 cation channels [43]. To assess these mechanisms, we applied a combination of heat (an increase of +3.5 °C from 35 °C) and 100 microM cyclic ADP-ribose (cADPR) to evaluate OT release capacity.

OT concentrations were measured over a 3 min period in dishes containing isolated hypothalamus, as previously described (Figure 6A) [43]. OT concentrations in the incubation medium at 35 °C without cADPR were 55.3 ± 6.6 pg/mL (*n* = 7) in *Slp*^+/+^ male mice and 56.5 ± 6.7 pg/mL (*n* = 7) in *Slp*^−/−^ male mice (Figure 6B). Under the stimulated condition, (with cADPR and temperature elevation from 35 °C to 38.5 °C), OT concentrations increased to 66.0 ± 6.4 pg/mL (*n* = 7) in *Slp*^+/+^ mice and 108.0 ± 17.2 pg/mL (*n* = 7) in *Slp*^−/−^ mice. OT release differed significantly between stimulated and unstimulated conditions (*n* = 7, t = 2.858, *p* = 0.0144; Figure 6C). The ratio of OT release in response to cADPR and heat was significantly greater in *Slp*^−/−^ mice (2.09 ± 0.29-fold) than in *Slp*^+/+^ mice (1.22 ± 0.09-fold; *n* = 8,9, t = 2.683, *p* = 0.0170; Figure 6D). The result indicates that CD38- and TRPM2-dependent OT release from the hypothalamus is more strongly enhanced in *Slp*^−/−^ male mice.

### 4.5. Further Movement and Performance Tests

**Figure 7:**
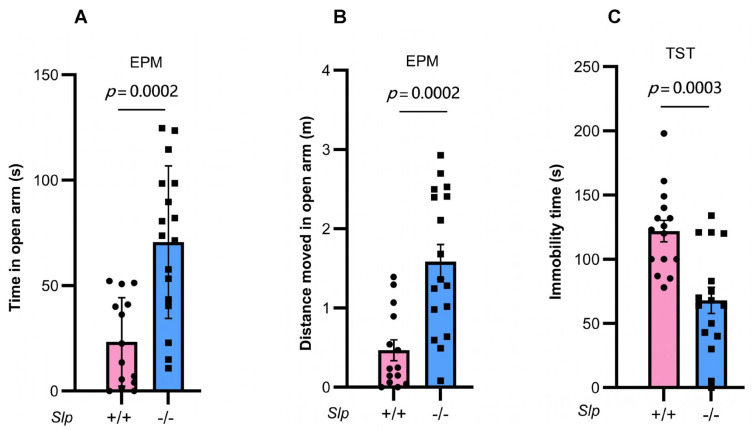
Male mouse behavior in the elevated plus maze (EPM) and tail suspension test (TST). Time (**A**) and distance moved (**B**) in the open arms during 10 min of the elevated plus maze test in *Slp*^+/+^ (pink) and *Slp*^−/−^ (blue) mice. (**C**) Immobility time during the tail suspension tests of *Slp*^+/+^ and *Slp*^−/−^ mice. *p* = 0.0002 or 0.0003, respectively. A dot represents an experimental result in one mouse.

Finally, we performed the EPM test, a widely validated paradigm for quantifying exploratory drive and anxiety in rodents [49]. *Slp*^−/−^ male mice stayed longer in the open arm (t = 1.021, *p* = 0.0002; Figure 7A) compared with the wild-type *Slp*^+/+^ mice, with significant increases in total distance (t = 4.189, *p* = 0.0002; Figure 7B), indicating reduced anxiety-related behavior and elevated locomotor activity.

To further validate the higher movement activity observed in the open arm of the EPM test in knockout mice, we examined mobility behavior under depression-like conditions using the tail suspension test. A significant decrease in immobility was observed in *Slp*^−/−^ male mice compared with *Slp*^+/+^ mice (*t* = 4.086, *p* = 0.0003; Figure 7C). These results may reflect altered arousal or motor activation rather than reduced anxiety or depressive-like behavior per se.

## 5. Discussion

**Figure 8:**
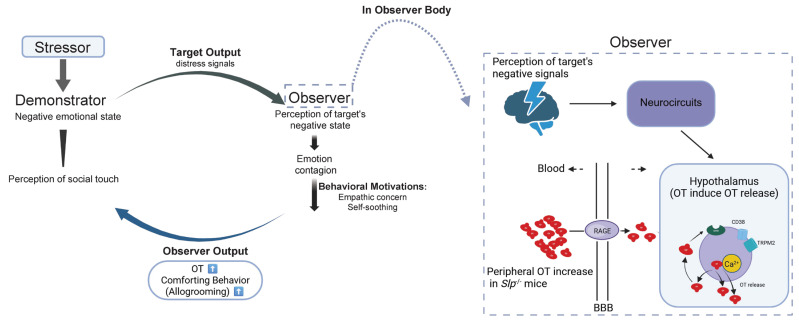
Schematic depicts the transfer of distress signals from demonstrator mice to observer mice and possible mechanisms underlying comforting behavior in observer mice. Molecular and cellular mechanisms underlying Increased oxytocin (OT, red) concentrations in the brain in observer mice after receiving distressed signals from demonstrator mice are schematized, resulting in increased comforting behavior in observer mice. During interaction at reunion, OT in observer mice increased via OT transport over the blood–brain barrier (BBB) via RAGE on neurovascular endothelial cells. In addition, OT release is facilitated by cADPR (CD38) and heat (TRPM2 channels), which is a part of the OT-induced OT release mechanism. These OT increases enhance recognition of the demonstrator’s distressed state in the demonstrator mice. Emotion to demonstrator mice in observer mice shapes comforting behavior (prosocial allogrooming). Green represents OT receptors.

The present study demonstrated that *C4a* knockout (*Slp*^−/−^) mice [28] exhibited hyperactive locomotion and reduced anxiety-like behavior. We also examined the same mouse line with deletion of one of the two complement component genes, the *C4A* and *C4B* genes [44]. Notably, *C4B* (also referred to as the *Slp* gene) is expressed exclusively in male mice in some strains. The resulting plasma concentrations of C4a were partially reduced in *Slp*^−/−^ mice because another *C4* gene was intact. The most striking behavioral finding is that in *Slp*^−/−^ mice, the observer *Slp*^−/−^ male mice displayed increased comforting allogrooming behavior toward demonstrators exposed to stressors, such as brief isolation from their old familiar cage and body restraint. The allogrooming behavior observed in *Slp*^−/−^ mice indicates that these animals can recognize their affiliative cage mates and that emotional stress in demonstrator mice may be transmitted to observers (Figure 8). The difference between groups that remained co-housed (familiar pairs) was also apparent compared with those that were separated prior to testing (non-familiar pairs). The findings underscore that the familiarity between observers and demonstrators is critical to display comforting behavior, because the single housing period was imposed at the end of cohousing, resulting in reduced allogrooming behavior. Future studies should explore whether other genetically or pharmacologically manipulated models of altered anxiety and social behavior similarly display heightened allogrooming. This would clarify whether the *Slp*^−/−^ phenotype probably reflects a unique complement-mediated enhancement of empathy-like behavior or a more generalizable mechanism involving social-affective processing.

The present findings suggest that higher locomotion with less anxiety and increased affiliative behavior remains unclear. However, such active behaviors may promote empathic feelings and allogrooming. In other studies, on the contrary, hyperactivity can also be associated with anxiety in socially stressed models [50]. The involvement of GABA or dopamine other than OT should be considered in allogrooming behavior.

In addition, we demonstrated higher c-Fos staining in OT-positive cells in *Slp*^−/−^ mice compared with *Slp*^+/+^ mice during social interaction at reunion following isolation, with or without body restraint stress. This result not only shows the role of C4a in blood circulation [28] but also newly suggests its role in the nervous system, although the current study did not directly manipulate C4a levels in the brain or measure neuronal C4a.

However, since the net change in activated neurons with c-Fos staining between body restraint conditions and non-restraint conditions was not large in *Slp*^−/−^ mice, these mice may not exhibit specific activation, which could be due to signal saturation.

Compelling evidence supporting this is that oxytocinergic neurons are sensitive to the cADPR- and heat-dependent OT release mechanism [43] in mice with low plasma C4a levels (*Slp*^−/−^ mice). This indicates that C4a contributes not only to plasma OT regulation [28] but also to neuronal activity in the brain. Therefore, based on the present results, it is a high possibility that the modulation of brain activity through C4a resulted in the promotion of social interaction, especially comfort behavior (Figure 8). Collectively, these results indicate that C4a modulation of hypothalamic OT signaling may directly facilitate affiliative and empathy-related behaviors, suggesting a novel neuroimmune role for complement components in social behavior regulation, warranting further mechanistic investigation.

It has been established that CD38 and TRPM2 cation channels mediate brain OT release by increasing the intracellular free Ca^2+^ concentrations [43]. NAD metabolites generated by CD38, including ADPR and cADPR, facilitate Ca^2+^ influx via TRPM2 channels and promote Ca^2+^ mobilization through ryanodine receptors on intracellular Ca^2+^ stores [25]. As previously described by Zhong et al. [43], we observed stimulus-induced OT release from whole hypothalamus samples dissected from *Slp*^+/+^ and *Slp*^−/−^ male mice in the current study. OT levels in the incubation medium were significantly elevated in *Slp*^−/−^ mice compared with *Slp*^+/+^ mice, indicating that the *Slp*^−/−^ hypothalamus exhibits heightened sensitivity of the OT release system, particularly the OT-induced OT release mechanism (Figure 8).

However, although the OT involved mechanism can be inferred in allogrooming behavior, the direct influence of brain C4a on neural activity and social behavior remains unclear. To address this, manipulation of brain C4a concentrations, either via upregulation or suppression including experimental strategies of viral overexpression or RNAi-mediated knockdown, may be required. Because C4a is expressed in neurons, astrocytes, and microglia [50], comforting information may be relayed among activated neurons in several brain regions (neurocircuits) such as the anterior cingulate cortex, bed nucleus of the stria terminalis, paraventricular nucleus (PVN), basal/basolateral and central nucleus of the amygdala, and lateral habenular nucleus [40,50]. Such a signal is then conveyed to the hypothalamus (Figure 8) [40].

The OT signaling system is a key mediator of allogrooming behaviors, as demonstrated in voles [42]. In monogamous voles, 1 week of co-housing is sufficient to induce allogrooming, while mice of the ICR strain required longer co-housing. Interestingly, in the current experiment, familiar mice were from the same litter and were housed together, while unfamiliar mice, which were distinguished only by a 10-day isolation, displayed reduction in allogrooming behavior. This point should be examined more carefully in the near future. Nonetheless, our findings demonstrate that ICR mice can detect emotional cues from distressed demonstrators (Figure 8).

In addition, immunohistochemical analyses revealed enhanced activation of hypothalamic oxytocinergic neurons in observer *Slp*^−/−^ mice during interactions with either control or stressed cage mate (demonstrator) mice. Affiliative (allogrooming) behavior was more frequent and extended in *Slp*^−/−^ mice. The upregulated activity of CD38 and TRPM2 in *Slp*^−/−^ mice could be one of the reasons, because *Slp*^−/−^ mice displayed affiliative behavior more than *Slp*^+/+^.

In humans, the OT system mediates responses to interpersonal stressors, including empathic concern (sympathetic) and distress-related responses in observers to an emotional video (demonstrating media) [51]. Notably, Barz et al. showed that CD38 genetic variation is associated with increased personal distress [51]. However, systematic measurement of C4a concentrations in human serum or saliva, coupled with behavioral or emotional assessments, is necessary to examine correlations between C4a levels and emotional states in clinical populations, such as schizophrenia, autism spectrum disorders, postpartum depression, and multiple sclerosis [29,30,31,32,33]. These investigations are currently underway. In addition, from the viewpoint of translational framing, affiliative traits and parental sensory processing sensitivity influence children’s attention to emotional stimuli [52]. This kind of study on children could provide parallel evidence that individual differences in affiliation and sensitivity shape responses to emotional signals [52].

## 6. Conclusions

Our results demonstrate that *Slp*^−/−^ observer mice exhibit a significant increase in prosocial behavior, specifically allogrooming, reflecting empathic recognition of the emotional state of *Slp*^−/−^ demonstrators, which were distressed by body restraint. Such behavior seems to reflect neuronal activation in the hypothalamus during reunion depending on the genotype. *Slp*^−/−^ demonstrator mice effectively transmit information regarding the stressor and their emotional condition to familiar observer mice. Increased OT based on lower C4a concentrations and OT released by CD38 and TRPM2 in the hypothalamus may induce comforting targeting (helping) behavior in observer mice, leading to stress buffering in recipient mice (Figure 8). These findings may provide novel insights into the role of OT in mediating prosocial behaviors, emphasizing its potential relevance for understanding social empathy in complex social systems.

## Data Availability

The datasets used and/or analyzed during the current study are available from the corresponding author on reasonable request due to the animal center’s policy.

## Supplementary Materials

The following supporting information can be downloaded at: https://www.mdpi.com/article/10.3390/brainsci16010081/s1, Table S1: Detailed statistical information; Video S1: supplementary movies.
