# Supplementary material for: Increase in Comforting Behavior (Allogrooming) During Social Interaction in Male Mice Deficient for the Slp Gene of Complement Component C4
Source: Brain Sci. 2026 Jan 7;16(1):81. doi: 10.3390/brainsci16010081 (PMC12838630; doi:10.3390/brainsci16010081)
Supplement: Supplementary file 1 [file brainsci-16-00081-s001.zip › brainsci-4041286-supplementary.pdf]

Supplementary Table S1. Detailed statistical information.			
Figure	Sample size	Statistical test	Test statistic and P value
Fig. 1B	n=17,14	Two-sided unpaired t test	t=3.792, P=0.0007
Fig. 1C	n=17,14	Two-sided unpaired t test	t=3.791, P=0.0007
Fig. 1D	n=17,14	Two-sided unpaired t test	t=2.168, P=0.0385
Fig. 1E	n=17,14	Two-sided unpaired t test	t=4.167, P=0.0003
Fig2	n = 10 male mice in each group	One-way ANOVA	F=8.495，P=0.0002
Fig. 3C	n = 10 male mice in each group	Two-way ANOVA, post hoc two-sided unpaired t test	ANOVA:<br>Measurement (control vs. restraint):F (1, 36) = 1.009 P=0.322<br>Genotype (*Slp*+/+ vs. *Slp* -/-):F (1, 36) = 16.92 P<0.001<br>Interaction: F (1, 36) = 1.009 P=0.322<br>Pairwise comparisons:<br>*Slp*+/+ without body restraint VS *Slp* -/- without body restraint: t=3.178, P=0.0052<br>*Slp*+/+ with body restraint VS *Slp* -/- with body restraint: t=2.622, P=0.0173<br>*Slp*+/+ without body restraint VS *Slp* +/+ with body restraint: t=10.42, P<0.0001<br>*Slp*-/- without body restraint VS *Slp* -/- with body restraint: t=5.812, P<0.0001
Fig. 3D	n=10,10,10,10,9,9	Two-way ANOVA, post hoc two-sided unpaired t test	ANOVA:<br>Measurement (control vs. restraint):F (2, 54) = 0.5441 P=0.5835<br>Treatment:F (1, 54) = 80.80 P<0.001<br>Interaction: F (2, 54) = 1.009 P=0.5441<br>Pairwise comparisons:<br>*Slp*-/- without body restraint VS *Slp* -/-

			with body restraint: t=3.178, P＜0.001<br>*Slp*-/- without body restraint VS *Slp* -/- without body restraint+OT: t=2.622, P=0.0043<br>*Slp*-/- without body restraint+Saline VS *Slp* -/- with body restrain+Salinet: t=2.456, P＜0.0001<br>*Slp*-/- without body restraint+Saline VS *Slp* -/- without body restraint+OT: t=2.652, P=0.0843<br>*Slp*-/- without body restraint+OT VS *Slp* -/- with body restrain+OT: t=2.687, P=0.0005
Fig. 3E	n = 9 male mice in *Slp* -/- OT and *Slp* -/- Saline group<br>n =7 male mice in *Slp* -/- OTA group	One-way ANOVA	F=8.728，P=0.0015
Fig.4B	n=5 in each group	Two-sided unpaired t test	*Slp*+/+ without body restraint + familiarity VS *Slp*+/+ without body restraint –familiarity: t=2.779, P=0.0148<br><br>*Slp*+/+ without body restraint – familiarity VS *Slp*+/+ with body restraint –familiarity: t=5.289, P=0.0007<br><br>*Slp*+/+ with body restraint + familiarity VS *Slp*+/+ with body restraint – familiarity: t=2.677, P＜0.0001<br><br>*Slp*−/− without body restraint + familiarity VS *Slp*−/− without body restraint –familiarity: t=2.271, P=0.0423<br><br>*Slp*−/− without body restraint -

			familiarity VS *Slp*−/− with body restraint –familiarity: t=5.478, P=0.0006<br><br>*Slp*−/− with body restraint + familiarity VS *Slp*−/− with body restraint –familiarity: t=1.055, P=0.3107
Fig. 5B	n = 8 male mice in each group	Two-way ANOVA	All has no significant
Fig. 5C	n = 8 male mice in each group	Two-way ANOVA	All has no significant
Fig.5D	n = 8 male mice in each group	Two-way ANOVA	Measurement (control vs. restraint):F (1, 29) = 12.40 P=0.0014<br>Genotype (*Slp*+/+ vs. *Slp* -/-):F (1, 29) = 23.18 P<0.0001<br>Interaction: F (1, 29) = 0.4885 P=0.4903
Fig. 6B	n=7	Two-sided unpaired t test	t=1.021 ,P=0.3272
Fig. 6C	n=7	Two-sided unpaired t test	t=2.858 ,P=0.0144
Fig. 6D	n=8，9	Two-sided unpaired t test	t=2.683,P=0.017
Fig. 7A	n=14,17	Two-sided unpaired t test	t=4.189,P=0.0002
Fig. 7B	n=14,17	Two-sided unpaired t test	t=4.189,P=0.0002
Fig. 7C	n=15,16	Two-sided unpaired t test	t=4.086,P=0.0003
